# Efficient generation of mouse ESCs-like pig induced pluripotent stem cells

**DOI:** 10.1007/s13238-014-0043-2

**Published:** 2014-03-27

**Authors:** Qi Gu, Jie Hao, Tang Hai, Jianyu Wang, Yundan Jia, Qingran Kong, Juan Wang, Chunjing Feng, Binghua Xue, Bingteng Xie, Shichao Liu, Jinyu Li, Yilong He, Jialu Sun, Lei Liu, Liu Wang, Zhonghua Liu, Qi Zhou

**Affiliations:** 1 State Key Laboratory of Reproductive Biology, Institute of Zoology, Chinese Academy of Sciences, Beijing, 100101 China; 2 College of Life Science, Northeast Agricultural University of China, Harbin, 150030 China; 3 Intelligent Polymer Research Institute, ARC Center of Excellence for Electromaterials Science, AIIM Facility, University of Wollongong, Wollongong, NSW 2522 Australia

**Dear Editor,**

Porcine induced pluripotency stem cells (piPSCs) are promised in basic research, animal husbandry and regenerative medicine. However, the efficiency of the piPSCs induction has been low and the generated piPSCs varied in cell morphology and cell characteristics. Here we report a novel approach to improve efficiency of piPSCs generation. The induced piPSCs are dome-shaped mouse embryonic stem cells (ESCs)-like and display molecular properties of mouse ESCs. Electroporation study reveals that mouse ESCs-like status facilitates genetic manipulating of piPSCs. Importantly, we demonstrate that the domed piPSC colonies are more suitable as donor cells for nuclear transfer (NT) to generate reconstructed embryos than those flattened piPSCs. The potential applications of the newly generated piPSCs in ungulate pluripotent research are discussed.

Previously, we generated the mouse ESCs-like human ESCs by modifying culture conditions (Gu et al., 2012). In this study, we generated the mouse ESCs-like piPSCs and enhanced induction rate of piPSCs by using LBX medium. The whole process of piPSCs generation was illustrated on Fig. 1A. The donor cells used for induction were pig embryonic fibroblasts (PEFs) that were obtained from 33.5 dpc embryos of Duroc strain. In LBX medium, the small colonies formed as early as the fifth day after induction, whereas, in KOSR medium, the colonies were firstly visualized at the eighth day after induction (Fig. 1B). We also tracked and compiled the number of colonies kinetically for five days and the efficiency of piPSCs formation in LBX medium was about five times of that in the KOSR medium (Fig. 1B). We then picked the colonies to derive some piPSCs lines. We compared piPSCs cultured in LBX medium with those cultured in KOSR medium and found some differences between them. Morphologically the former colony looked domed, just like mouse ESCs (these piPSCs were called pips_m cells), whereas, the later looked flattened (these piPSCs were called pips_h cells) (Fig. 1C). These cells also differed in cell viability and proliferation ability. The cells cultured in LBX medium had higher proliferation ability than those cultured in KOSR medium (Fig. 1D). Consistent with this observation, we also found that the capability of single cell colony formation was much stronger in LBX medium (Fig. 1E). The pips_m cells were predominantly diploid with normal 38 chromosome karyotypes (Fig. S1C) and expressed normal level of nuclear pluripotent marker OCT4 and membrane markers SSEA4, TRA-1-60 and SSEA1 (Fig. 1F), however, the pips_h cells did not express SSEA1 (Fig. S1A). We further detected the differentiation ability of these cells. Both types of the piPSCs could form embryonic bodies (EBs) *in vitro* when cultivated in suspension. The EBs also expressed markers of three germ layers (Fig. 1G) and had the neuron differentiation ability (Fig. 1H). The piPSCs could form teratomas when injected into immune-deficient mouse, and the teratomas were comprised of tissues from three germ layers (Fig. 1I). The detection of X chromosome status for both type of piPSCs derived from the same female fibroblasts were carried out by H3K27 trimethyl staining, and we found that the most of pips_h cells had one inactive X chromosome. As expected, both X chromosomes were active in most of pips_m cells (Fig. S2).

**Figure 1:**
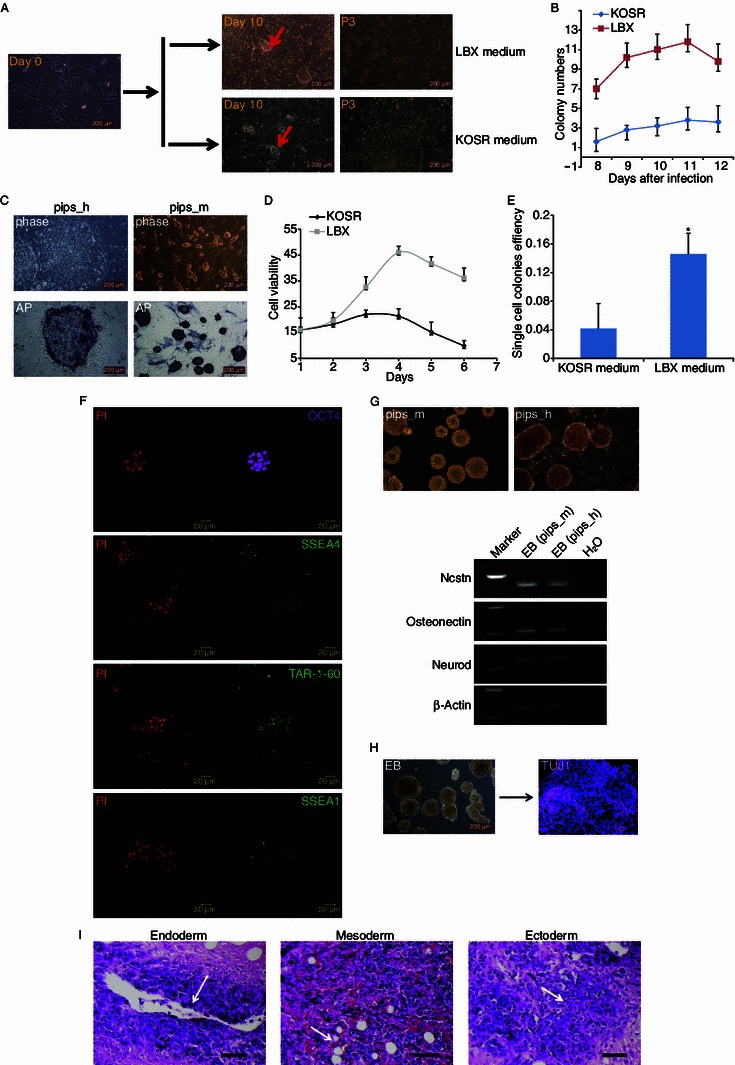
**Efficient generation of mouse ESCs-like piPSCs in LBX medium**. (A) Timeline for generating piPSCs in LBX and KOSR medium. Day 0 shows PEFs before infected with OSKM retrovirus, on the second day the infected PEFs were plated on MEF feeders with LBX and KOSR mediums. Day 10 shows PEFs infected 10 days later and colonies were yielded (red arrows). In LBX medium the colonies looked domed and were picked for digesting to derive cell lines. In KOSR medium the colonies looked flattened and were picked for cutting to derive cell lines. P3 shows the cell lines in KOSR and LBX mediums had been passaged for 3 times. Scale bars are 200 μm. (B) Colony number. Colonies were counted from 8 to 12 days after infection in LBX and KOSR medium, respectively. (C) Top, phase morphology of piPSCs derived in KOSR (pips_h) and LBX (pips_m) mediums. Bottom, piPSCs clones were stained with alkaline phosphatase kit. Scale bars are 200 μm. (D) Cell viability analysis for different cell lines. KOSR and LBX show the cell viability in KOSR and LBX medium. (E) Single cell colony formation. Cell lines derived in LBX medium had higher colony formation ability than that derived in KOSR medium. *P < 0.05. (F) Pips_m cells clones were stained with pluripotency markers. Positive OCT4 (purple), SSEA4 (green), TRA-1-60 (green) and SSEA1 (green) were observed. DNA was stained with propidium iodide (PI, red). Shown were examples from pips-n-3. Scale bars are 20 μm. (G) *In vitro* embryoid body formation. Top, pips_m and pips_h cells (from pips-n-3 and pips-5 respectively) could form EB both, scale bars are 200 μm. Bottom, endoderm (*Ncstn*), mesoderm (*Osteonectin*) and ectoderm (*Neurod*) markers were detected by RT-PCR. (H) Directed differentiation of pips_m cell derived EBs into neural linage. Left, EBs from examples of pips-n-3. Right, differentiated neurons were stained with Tuj1 (purple). DNA was stained with Hoechst (blue). (I) Teratoma formation of pips_m cells (from pips_m cell line pips-n-3). Tissues exhibiting all three germ layers were presented on the teratoma dissection slices identified by staining with haematoxylin and eosin. Left, endoderm with glands; Middle, mesoderm with fat tissues; Right, ectoderm with nervous tissues. Scale bars are 500 μm

Through Q-PCR analysis, we found that both the pips_m and pips_h cell lines expressed *Oct4* and *c-Myc* transgenes (*Oct4_v* and *c-Myc_v*), but the expression pattern of the transgenic *Sox2* and *Klf4* (*Sox2_v* and *Klf4_v*) were different between the pips_m and pips_h cell lines. The latter did not express *Sox2_v* but expressed *Klf4_v* continuously. The former had perpetual expression of *Sox2_v* and *Klf4_v* (Fig. S3A). The expression of these four endogenous genes had also been detected. The expression of *Oct4* and *c-Myc* (*Oct4_3*′*UTR* and *c-Myc_3*′*UTR*) were increased in all the lines of piPSCs, as compared with the original PEFs, but was lower than that of transgenic ones (Fig. S3A and S3B). As the methylation status of Oct4 promoter region is relevant to the gene expression of Oct4, the methylation status of promoter region of Oct4 were evaluated (Fig. S3C). Compared to the parthenogenetic blastocyst embryos (PA_Blastocyst), the methylation of Oct4 promoter in the pips_h and the pips_m cells was not similar to that in PA_Blastocyst. In addition to *Oct4_3*′*UTR* and *c-Myc_3*′*UTR*, endogenous *Nanog* and *Sox2* (*Nanog_3*′*UTR* and *Sox2_3*′*UTR*) were measured as well. There was no difference in *Sox2_3*′*UTR* expression between pips_h and pips_m cells. Unexpected, the *Nanog_3*′*UTR* expression was higher in pips_h cells than that in pips_m cells (Fig. S3B). Because the pips_h cells appeared similar to human ESCs colonies, whereas the pips_m cells looked like mouse ESCs colonies, we further defined the differences in signaling pathway between them. The expression of *Lif*, *Lifr* and *Bmp4* were higher in the pips_m cells than that in the pips_h cells, *Smad4* and *Fgf2r* expression were also higher in the pips_m cells than that in the pips_h cells (Fig. S3D). As with previously reported, the piPSCs expressed exogenous transgenic RNA. This was also the barrier of piPSCs development. There were no standard porcine ESCs generated up to now, so the research groups including us couldn’t define what the piPSCs should be like. We tried hard to optimize the system to generate “better” piPSCs for application.

Stem cells are ideal donors for generation of transgenic animals by NT due to their ability of self-renewal and high resolution screening for positive genetically modified cells (Zhou et al., 2010). Up to now, no healthy cloned adults had been generated directly from piPSCs, but, differentiated piPSCs or small molecular compounds treated-piPSCs could support *in vivo* development of embryos and live-born (Fan et al., 2013b). We compared the development potency of pig NT embryos reconstructed from the pips_m and pips_h cells both *in vitro* and *in vivo*. The rate of *in vitro* blastocyst development was higher in the pips_m cell group compared to the pips_h cell group (Table S1). Some differences were also noted *in vivo* development. The surrogate mothers into whom the pips_h cell-derived embryos were introduced were not pregnant, whereas there were three pregnant receipts from four pips_m cell lines (Table S2). These findings are in agreement with recent reports that mouse ESCs-like state piPSCs could contribute to pig embryonic development (Fujishiro et al., 2013).

Importantly, the pips_m cells demonstrated good cell viability and could be digested into single cells and passaged easily. Therefore they were suitable for transgenic application. We electroporated the pips_m and pips_h cells equally with the modified PiggyBac vector carrying red fluorescence protein (RFP) and neomycin-resistant (neo^r^) genes (Fig. S4A). Four days after the neomycin-resistant colonies appeared. Our results showed that the number of colonies yielded from the pips_m cells were 40 folds higher than that yielded from the pips_h cells with the same amount of the vector (Table 1). In addition, more colonies for the pips_h cells were noted after electroporation treated with Y27632 (Rock inhibitor). We randomly picked 30 pips_m neomycin-resistant colonies and 26 of them had been expanded to become cell lines (Fig. S4B). We also picked equal number of pips_h neomycin resistant colonies, however, only four colonies formed cell lines (Fig. S4C). Therefore the pips_m might have higher potential in transgene application.

**Table 1:** Electroporation of transgenes into pips_m and pips_h cells

Cell lines		Cells for electroporation	Electroporation	Average colonies after drug selection
pips_m	pips_n_3	1.1 × 10^6^	3	>4400
	pips_n_5	1.2 × 10^6^	3	>4580*
	pips_n_6	1.0 × 10^5^	2	>580
	pips_n_7	6.4 × 10^5^	3	
pips_h	pips_1	1.1 × 10^6^	3	120
	pips_2	1.2 × 10^6^	3	500*
	pips_5	1.0 × 10^5^	3	40
	pips_6	6.4 × 10^5^	3	

* Cells were treated with Y27632 after electroporation

High efficiency and availability of pluripotent stem cells are essential for their application in domestic ungulates. Some new genes had been involved to enhance piPSCs induction (Fan et al., 2013a; Petkov et al., 2013). In this work, we produced the pips_m cells with high efficiency by optimizing the culture medium. We concluded that the small molecular compounds in LBX medium contributed to the formation of mouse ESCs like colonies and improved cell viability. It was reported that these small molecular compounds had been used for converting human ESCs into mouse ESCs-like state and can maintain long-term survival of human ESCs (Hanna et al., 2010; Gu et al., 2012). After removing the small compounds from LBX medium, the colonies had a tendency to become flattened (Wang et al., 2013), suggesting that these small molecular compounds are responsible for mouse ESCs-like colony formation and cell proliferation. Another report also showed that a small molecular compound, Forskolin, could contribute to the generation of the naïve state of piPSCs (Fujishiro et al., 2013). During our work progress, other independent studies also found LIF and some inhibitors could modulate the pluripotency of piPSCs (Cheng et al., 2012; Cloke et al., 2012; Kwon et al., 2013). Nonetheless, this was a novel way to derive piPSCs and also had a comprehensive comparison of piPSCs for the two states. In our study, the pips_m cells had higher transgenic efficiency which indicated that the mouse ESCs-like state facilitated the transgenic manipulation (Buecker et al., 2010). As human ESCs morphologically were more similar to mouse EpiSCs, many studies had paid attention to the pluripotency of human ESCs and to converting human ESCs to mouse ESCs-like (Gu et al., 2012). Because of the ethical limitation, it was not possible to perform embryonic experiments to determine the pluripotency of human ESCs and to find that the converted human ESCs were prone to mouse ESCs at both phenotype and internality levels (Li and Ding, 2011; Young, 2011). Our results involved pluripotent research in other species. Domed colonies of piPSCs might have better developmental potency *in vitro* and *in vivo* than the flattened ones.

In summary, we were able to efficiently produce mouse ESCs-like piPSCs. The piPSCs had good cell viability and proliferation capability. Exogenic pancreas had been successfully generated using blastocyst complementation in pigs (Matsunari et al., 2013). Our study will provide a novel tool for further transgenic application and naïve states exploration of pig pluripotent cells.

## Electronic supplementary material

Below is the link to the electronic supplementary material.

Supplementary material 1 (PDF 913 kb)

## References

[CR1] Buecker C, Chen HH, Polo JM, Daheron L, Bu L, Barakat TS, Okwieka P, Porter A, Gribnau J, Hochedlinger K (2010). A murine ESC-like state facilitates transgenesis and homologous recombination in human pluripotent stem cells. Cell Stem Cell.

[CR2] Cheng D, Guo Y, Li Z, Liu Y, Gao X, Gao Y, Cheng X, Hu J, Wang H (2012). Porcine induced pluripotent stem cells require LIF and maintain their developmental potential in early stage of embryos. PLoS ONE.

[CR3] Cloke T, Munder M, Taylor G, Müller I, Kropf P (2012). Modulation of pluripotency in the porcine embryo and iPS cells. PLoS ONE.

[CR4] Fan A, Ma K, An X, Ding Y, An P, Song G, Tang L, Zhang S, Zhang P, Tan W (2013). Effects of TET1 knockdown on gene expression and DNA methylation in porcine induced pluripotent stem cells. Reproduction.

[CR5] Fan N, Chen J, Shang Z, Dou H, Ji G, Zou Q, Wu L, He L, Wang F, Liu K (2013). Piglets cloned from induced pluripotent stem cells. Cell Res.

[CR6] Fujishiro SH, Nakano K, Mizukami Y, Azami T, Arai Y, Matsunari H, Ishino R, Nishimura T, Watanabe M, Abe T (2013). Generation of naive-like porcine-induced pluripotent stem cells capable of contributing to embryonic and fetal development. Stem Cells Dev.

[CR7] Gu Q, Hao J, Zhao XY, Li W, Liu L, Wang L, Liu ZH, Zhou Q (2012). Rapid conversion of human ESCs into mouse ESC-like pluripotent state by optimizing culture conditions. Protein Cell.

[CR8] Hanna J, Cheng AW, Saha K, Kim J, Lengner CJ, Soldner F, Cassady JP, Muffat J, Carey BW, Jaenisch R (2010). Human embryonic stem cells with biological and epigenetic characteristics similar to those of mouse ESCs. Proc Natl Acad Sci USA.

[CR9] Kwon DJ, Jeon H, Oh KB, Ock SA, Im GS, Lee SS, Im SK, Lee JW, Oh SJ, Park JK (2013). Generation of leukemia inhibitory factor-dependent induced pluripotent stem cells from the Massachusetts General Hospital miniature pig. BioMed Res Int.

[CR10] Li W, Ding S (2011). Human pluripotent stem cells: decoding the naive state. Sci Transl Med.

[CR11] Matsunari H, Nagashima H, Watanabe M, Umeyama K, Nakano K, Nagaya M, Kobayashi T, Yamaguchi T, Sumazaki R, Herzenberg LA (2013). Blastocyst complementation generates exogenic pancreas in vivo in apancreatic cloned pigs. Proc Natl Acad Sci USA.

[CR12] Petkov S, Hyttel P, Niemann H (2013). The choice of expression vector promoter is an important factor in the reprogramming of porcine fibroblasts into induced pluripotent cells. Cell Reprogram.

[CR13] Wang J, Gu Q, Hao J, Jia Y, Xue B, Jin H, Ma J, Wei R, Hai T, Kong Q (2013). Tbx3 and Nr5alpha2 play important roles in pig pluripotent stem cells. Stem Cell Rev.

[CR14] Young RA (2011). Control of the embryonic stem cell state. Cell.

[CR15] Zhou S, Ding C, Zhao X, Wang E, Dai X, Liu L, Li W, Liu Z, Wan H, Feng C (2010). Successful generation of cloned mice using nuclear transfer from induced pluripotent stem cells. Cell Res.

