# Supplementary material for: Efficient generation of mouse ESCs-like pig induced pluripotent stem cells
Source: Protein Cell. 2014 Mar 27;5(5):338–42. doi: 10.1007/s13238-014-0043-2 (PMC3996154; doi:10.1007/s13238-014-0043-2)
Supplement: Supplementary file 1 — Supplementary material 1 (PDF 913 kb) [file 13238_2014_43_MOESM1_ESM.pdf]

Supplementary Materials

Materials and methods

Reagents and media

The reagents and basic media used in this study were purchased from Life Technologies. All small molecular compounds were purchased from Stemgent, Inc unless otherwise stated. The porcine embryonic fibroblast (PEF) of Duroc and Landrace pig were provided by Beijing Farm Animal Research Center. The recipient pigs for embryo transfer and the ICR mice for isolation of mouse embryonic fibroblast (MEF) were purchased from Beijing Vital River Company. All animals used in this study were handled according to the Guidelines of the Care and Use of Laboratory Animals established by Beijing Association for Laboratory Animal Science.

Cell culture

The KOSR medium consist of 76% Knockout Dulbecco's Modified Eagle's Medium (KO-DMEM, Invitrogen), 20% knockout serum replacement (Knockout SR, Invitrogen), 8 ng/ml bFGF (R&D), 2 mM L-glutamine (Invitrogen), 0.1 mM β-mercaptoethanol (Sigma), 1% MEM nonessential amino acids (NEAA, Invitrogen), and 1% penicillin-streptomycin (SP, Invitrogen). LBX medium contains mall molecular compounds including Erk inhibitor PD0325901(PD, 1 uM), Gsk3 inhibitor CHIR99021 (CH, 3 μM), activin receptor inhibitor SB431542 (SB, 2 μM) and ascorbic acid (VC, 50 ng/ml) as described previously (Gu et al., 2012). DMEM medium consist of 88% high glucose DMEM, 10% fetal bovine serum (FBS, Invitrogen), 1% NEAA, 2 mM L-glutamine, and 1% SP.

PEFs was obtained from 33.5 dpc pig embryos, and enzymatically digested by collagenase IV. They were cultured in DMEM medium and passaged using 0.25% Trypsin-EDTA (Invitrogen). The culture system of pips_h cells was similar to that of human ESCs(Hao et al., 2009). Pips_m cells were cultured in LBX medium and digested with TrypLE™ (Invitrogen). Both the porcine and mouse cells were cultured in a humid incubator at 37°C and 5% CO_2 (Thermo).

Retroviral transduction and infection

Retroviral transduction and infection were performed according to previously published protocol (Aasen et al., 2008). In brief, four mouse retroviral vectors, pMXs-Oct4, Sox2, Klf4 and c-Myc, were purchased from Addgene. A 100-mm dish of 293T cells were transfected with 12 μg retroviral vectors, 4 μg VSV-G and 8 μg Gag-Pol vector (with LTX regents) according to the manufacturer's protocol (Invitrogen). 12 h later, the medium was replaced by

DMEM containing 1% FBS, 1%NEAA, 2 mM L-glutamine, and 1% SP. The supernatant was harvested after 36 h and 60 h, respectively. They were filtered through 0.45 μm cellulose acetate filter (Millipore), and concentrated by centrifugal filter at 4000 rpm for 30 min. Finally, 10^4 PEFs per well were infected with concentrated virus in a 24-well plate. When the PEFs reached 90% confluence, they were split into 12-well plates pre-seeded with feeders. On the second day, the media were replaced by KOSR or LBX.

Immunofluorescence analysis and alkaline phosphatase staining

The piPSCs were fixed with 4% paraformaldehyde at room temperature for 10 minutes, permeabilized with 0.5% Triton X-100 at 37 ℃ for 10 minutes and blocked in 2% bovine serum albumin (BSA). The cells were incubated with the primary antibodies against OCT4 (Santa Cruz), NANOG (abnova), neuronal Class III β-Tubulin (Chemicon, Tuj1), H3K27 trimethyl (Millipore, H3K27 me3)(Shen et al., 2008), SSEA4 (Millipore), SSEA1 (Millipore) and TRA-1-60 (Millipore) at 4℃ overnight. The second day, followed by rinsing with PBS three times, the cells were incubated with Alexa Fluor secondary antibody (Jackson) at 37 ℃ for 1 hour. Finally, DNA was stained with propidium iodide (10 μg/ml; Molecular Probes, OR) or Hoechst 33342 (10 μg/ml, Invitrogen). Alkaline phosphatase (AP) staining was performed with BCIP/NBT Alkaline Phosphatase Colour Development Kit (Beyotime) according to the manufacturer's instructions.

Transgenic manipulation of piPSCs

When carrying out transgenic manipulation, the piPSCs were digested into single cells. 1×10^6 cells were electroporated with 6 μg plasmid carrying a PGK–neo^r cassette and 2 μg plasmid carrying a transposase cassette by Neon Transfection System (Invitrogen) at 2400 V, 20 ms and two pulses according to the manufacturer's instructions. Then the cells were plated onto 60-mm dishes coated with G418-resistant feeders and filled with the culture medium containing 250 μg/ml G418 for selection of resistant cells. After 4-6 days, G418-resistant colonies were selected for sub-cell-line derivation.

Karyotype analysis

Karyotype analysis was performed as previously described (Zhao et al., 2009).

Embryoid body formation

PiPSCs were digested into single cells and transferred into gelatin-coated plates followed by incubating in DMEM medium for 10 min to discard the feeder cells. Then the suspensions were harvested and transferred to a dish containing KOSR medium lacking bFGF. The

medium was changed every other day and on day 6 the EBs were collected.

Teratoma formation and histological analysis

After digesting, the piPSCs were transferred to gelatincoated plates to discard the feeder cells. Then, collect the cells for centrifuge. Next, suspend the cell pellets using PBS and 300 μl suspension containing 1×10^7 piPSCs were injected under the skin of non-obese diabetic/severe combined immune-deficient (NOD/SCID) mice. The teratomas could be harvested for histological analysis at 4 weeks. And then the tumors are fixed and sliced. Sections were stained with haematoxylin and eosin.

RT-PCR and quantitative PCR analyses

Total RNA was extracted using Trizol reagent (Invitrogen). RT-PCR was performed by using Q-PCR SYBR Green Real-time PCR Master Mix (Toyobo), High-Capacity cDNA Reverse Transcription Kits (Applied Biosystems, 4368814) and Real-time PCR equipment (Agilent Mx3005P) according to manufacturer's instructions. Electrophoresis was performed on a 2% agarose gel. All primers were listed in Table S3.

Statistical Analysis

PiPSCs colony numbers and quantitative PCR results were analyzed by analysis of variance (ANOVA) with $P< 0.05$ considered significant.

Reference

Aasen, T., Raya, A., Barrero, M.J., Garreta, E., Consiglio, A., Gonzalez, F., Vassena, R., Bilic, J., Pekarik, V., Tiscornia, G., *et al.* (2008). Efficient and rapid generation of induced pluripotent stem cells from human keratinocytes. Nat Biotechnol 26, 1276-1284.

Gu, Q., Hao, J., Zhao, X.Y., Li, W., Liu, L., Wang, L., Liu, Z.H., and Zhou, Q. (2012). Rapid conversion of human ESCs into mouse ESC-like pluripotent state by optimizing culture conditions. Protein Cell 3, 71-79.

Hao, J., Zhu, W., Sheng, C., Yu, Y., and Zhou, Q. (2009). Human parthenogenetic embryonic stem cells: one potential resource for cell therapy. Sci China C Life Sci 52, 599-602.

Shen, Y., Matsuno, Y., Fouse, S.D., Rao, N., Root, S., Xu, R., Pellegrini, M., Riggs, A.D., and Fan, G. (2008). X-inactivation in female human embryonic stem cells is in a nonrandom pattern and prone to epigenetic alterations. Proceedings of the National Academy of Sciences 105, 4709-4714.

Zhao, X.-y., Li, W., Lv, Z., Liu, L., Tong, M., Hai, T., Hao, J., Guo, C.-l., Ma, Q.-w., Wang, L., *et al.* (2009). iPS cells produce viable mice through tetraploid complementation. Nature 461, 86-U88.

Table S1 *In vitro* development of nuclear transfer embryos reconstructed from pig iPSCs

pig iPSCs cell lines		NO. of reconstructed embryos	No. of cleavage (%)	No. of blastocyst (%)
pips_h	pips-4	130	70 (53.28±4.19)[a]	8 (6.14±0.17)[c]
	pips-1	708	155 (53.42±8.40)[a]	19 (6.36±1.90)[c]
	pips-2	404	92 (61.10±7.31)[a]	14 (9.30±4.33)[c]
pips_m	pips-n-3	435	190 (80.2±5.37)[b]	26 (13.6±3.4)[d]
	pips-n-4	430	256 (59.05±5.83)[a]	64 (15.25±2.27)[d]
	pips-n-6	280	146 (52.00±3.03)[a]	28 (9.92±2.01)[d]
control	PEF	262	155 (84.2±5.03)[b]	38 (24.7±4.6)[e]

Note: Values with different superscripts indicate the numbers in the same column are significantly different and values with same superscripts indicate the numbers in the same column are not significantly different by one-way ANOVA ($p<0.05$)

Table S2 *In vivo* development of nuclear transfer embryos reconstructed from pig iPSCs

pig iPSCs cell lines		NO. of transferred embryos	No. of recipients	No. of pregnancies	No. of cloned piglets
pips_h	pips-1	270	2	0	0
	pips-2	310	2	0	0
pips_m	pips-n-3	630	4	2	0
	pips-n-4	280	1	1	0
	pips-n-6	696	4	0	0
	pips-n-10	1074	3	2	0

Table S3 Primer sequence for RT-PCR and Q-PCR

Gene name	Forward primers	Reverse primers
Oct4_3'UTR	CAAACTGAGGTGCCTGCCCTTC	ATTGAACTTCACCTTCCCTCCAACC
Sox2_3'UTR	CATCAACGGTACACTGCCTCTC	ACTCTCCTCCCATTTCCCTCTTT
Nanog_3'UTR	AATGATCGTCACATATCTTCAGGCTGTA	GTTCCATGGGCTCAGTGGTCAAG
C-myc_3'UTR	ATCCAAGACCACCACCACTG	GTTCACAGCAACATTCAGGTAGA
Oct4_v	GAGGCCCTTGGAAGCTTAGCC	CCCAGTGTGGTGGTACGGGAAATC
Sox2_v	TTGACGCGGTCCGGGCTGTTCT	CCCAGTGTGGTGGTACGGGAAATC
Klf4_v	TAGTCGGGGCACCTGCTGGACGC	CCCAGTGTGGTGGTACGGGAAATC
C-myc_v	CGCTCTGCTGTTGCTGGTGAT	CCCAGTGTGGTGGTACGGGAAATC
Fgfr1	ACTGCTGGAGTTAATACCACCG	GCAGAGTGATGGGAGAGTCC
Lif	CACTGGAAACACGGGGCA	AGGGCGGGAAGTTGGTCA
Bmp4	CGTCATCCCAGATTACAT	GAGTCGAAGCTCTGCGGAT
Smad4	GGCTTCAGGTGGCTGGTCGGA	ACCTGATGGAGCATTACT
Lifr	CTCATCCCAGTGGCAGTG	CCAGAACCTCAACATTAT
bfgf	GCGACCCTCACATCAAACT	CAGTGCCACATACCAACT
Ncstn	CAGCAAAGAACTGGAGTTCATCACTCT	AGGAAAAGCTGGGGTCCTCTTCAG
Osteonectin	TCGGCATCAAGGAGCAGG	CAGGCAGAACGACAAACCAT
Neurod	GACGAATGAAAGCCAACG	CTCCGACAGAGCCCAGAT
β-Actin	AGATCGTGCGGGACATCAAG	GCGGCAGTGGCCATCTC

Supplementary Figure Legends

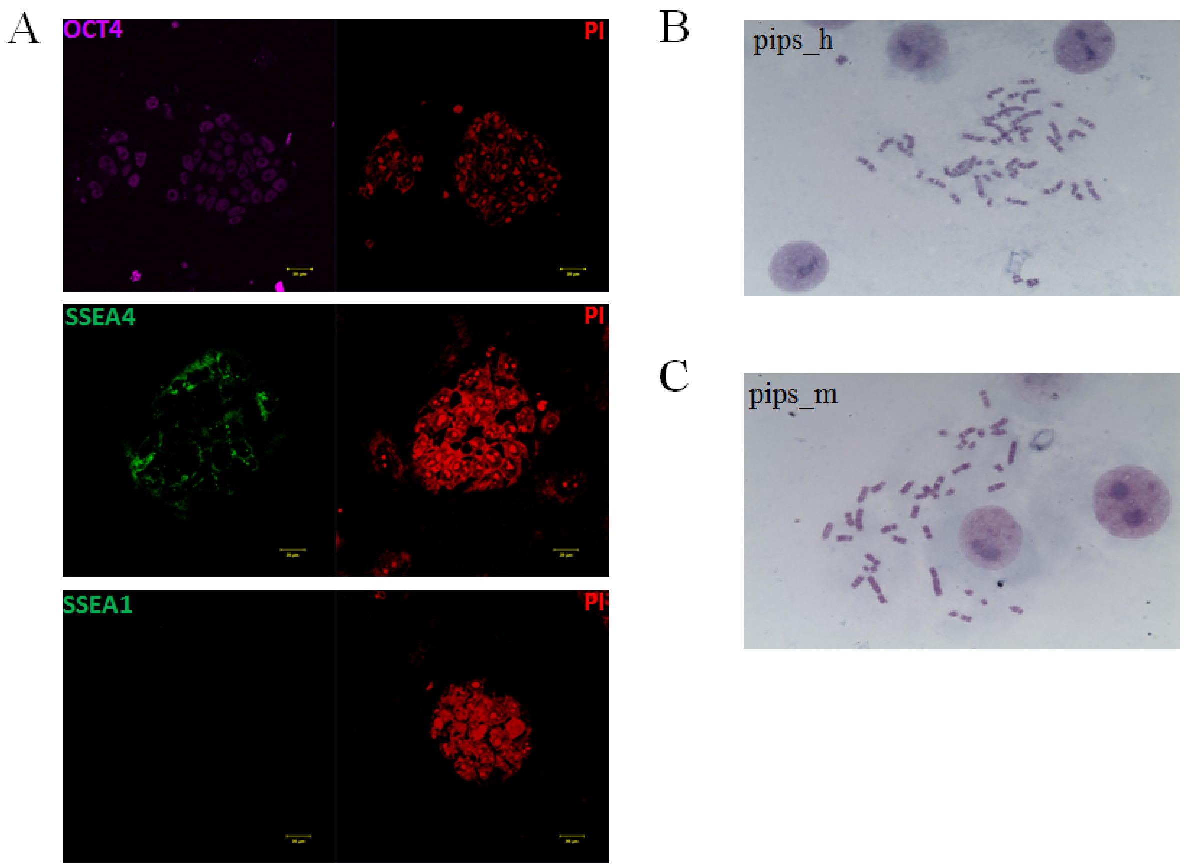

Figure S1 |Immunostaining for pluripotency markers in pips_h cells and karyotype analysis

(A) Positive OCT4 (purple) and SSEA4 (green) were observed. SSEA1 was negative in pips_h cells. The nucleus was stained with PI (red). Shown are examples from the pips-2 line. Scale bars are 20 µm.

(B) Karyotype analysis of pips-h cells. Shown here is a pips-1 spread. More than 75% of cells showed normal pig karyotype of 38 chromosomes.

(C) Karyotype analysis of pips-m cells. Shown here is a pips-n-6 spread. More than 75% of cells showed normal pig karyotype of 38 chromosomes.

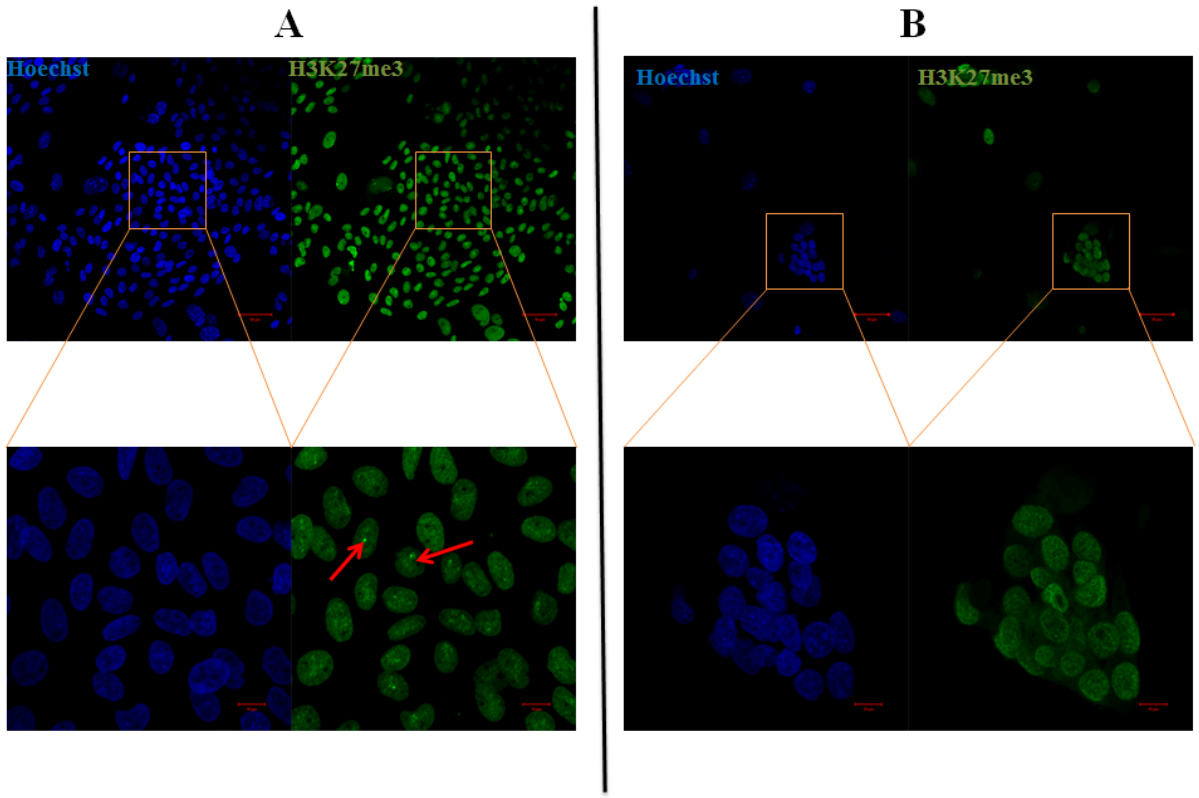

Figure S2 | X chromosome status of pips_h and pips_m cells

(A) Pips_h cells clones were stained with H3K37 trimethyl (H3K27me3) antibodies. Red arrow pointed the H3K27me3 'spot'. Hoechst was used to stain the nucleus. Shown are examples from the pips-5 line. Top bars are 50 µm. Bottom bars are 10 µm.

(B) Pips_m cells clones were stained with H3K37 trimethyl (H3K27me3) antibodies. There were no H3K27me3 'spot'. Hoechst was used to stain the nucleus. Shown are examples from the pips-n-3 line. Top bars are 50 µm. Bottom bars are 10 µm.

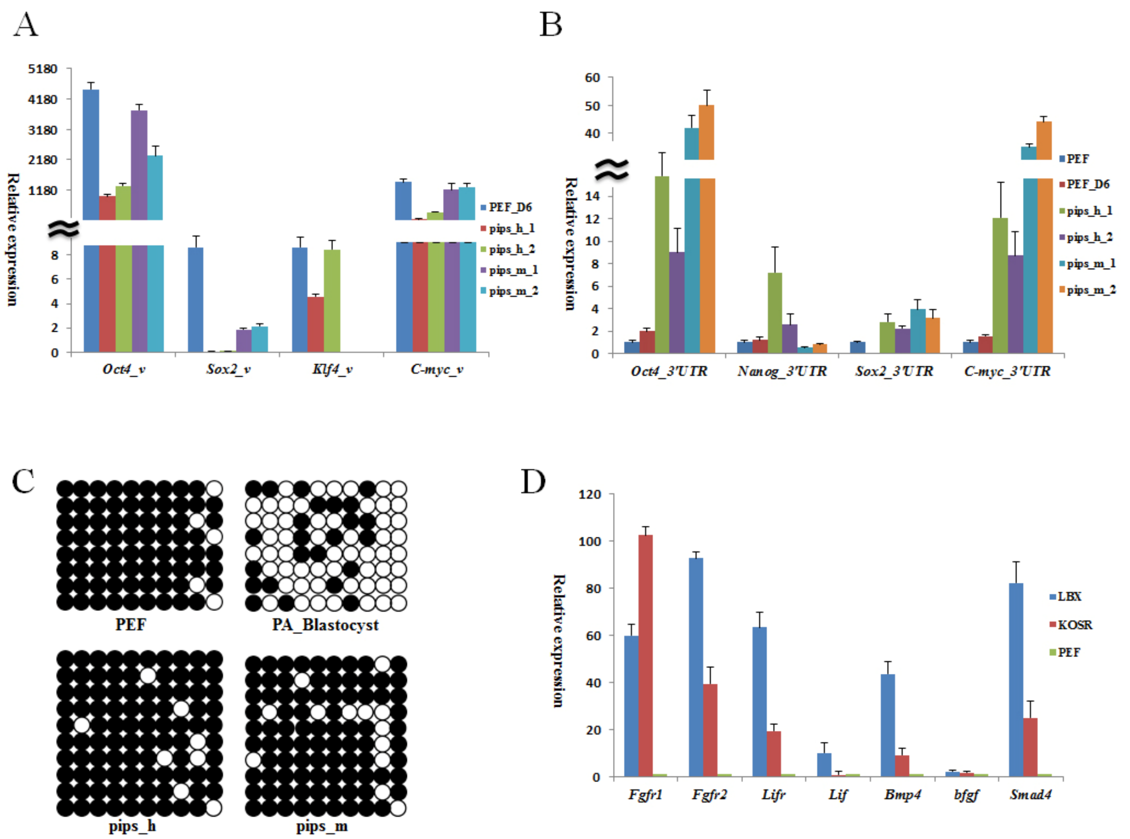

Figure S3 |Pips_m cells had different genes expression from pips_h cells

(A) Pips_m and pips_h cells had different transgene expression patterns. *Oct4_v* and *c-Myc_v* had not been silenced in pips_h and pips_m cells. In pips_m cells, *Klf4_v* had been silenced and *Sox2_v* remained active but pips_h cells had reverse patterns of *Klf4_v* and *Sox2_v* compared to pips_m cells. Positive control was the PEFs infected after 6 days (PEF_D6). Gene expression values were normalized with respect to β-actin.

(B) Endogenous expression assays of pips_m and pips_h cells. There were specific primers to discriminate endogenous expression. *Oct4_3'UTR* and *c-Myc_3'UTR*expression was higher in pips_m and pips_h cells than that in PEF_D6 and normal PEFs, but that was lower than the comprehensive transgenic expression. Pips_m and pips_h cells both had same level of *Sox2_3'UTR* expression, which was higher than PEF_D6 and normal PEFs. Pips_h cells had higher endogenous *Nanog* expression than pips_m cells. Gene expression values were normalized with respect to β-actin.

(C) Bisulfite sequencing of *Oct4* promoter for pips_m and pips_h cells. Negative control was PEF and positive control was porcine parthenogenetic blastocyst embryos (PA_Blastocyst).

(D) Q-PCR analysis of some signaling gene expression in pips_m and pips_h cells. Gene expression values were normalized with respect to β-actin.

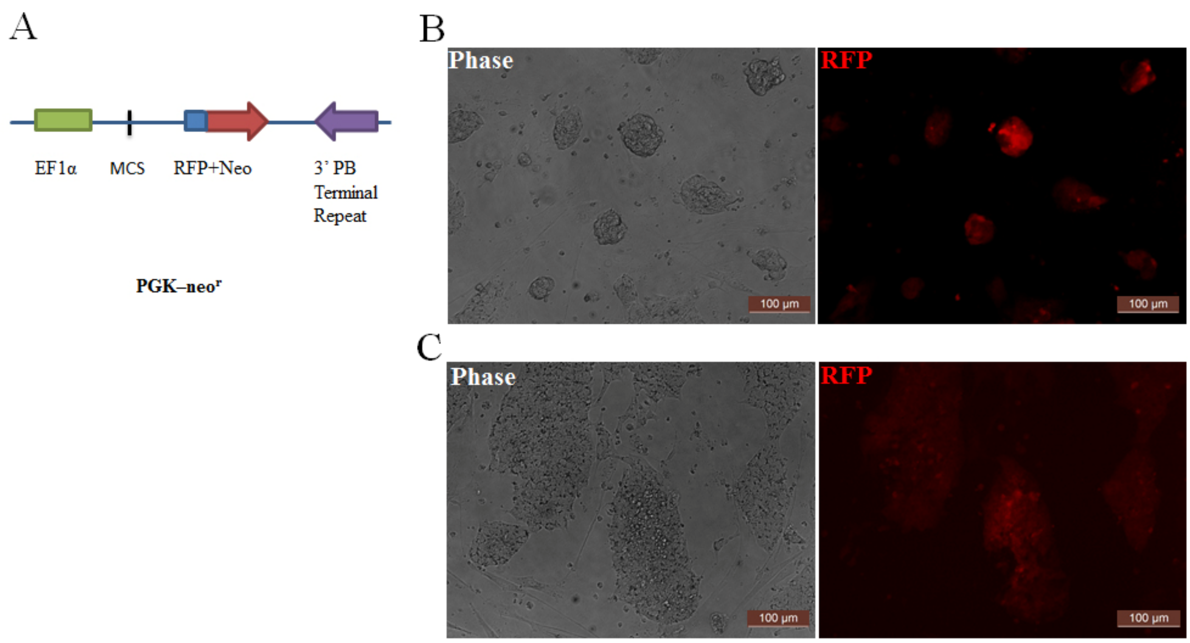

Figure S4 |Transgenic application of pips_m cells

(A) A diagram of modified PiggyBac vector (PGK–neor). RFP and neo^r loci were shown.

(B) The pips_m sub-cell-line from picked RFP positive clones. Left, bright filed image. Right, red fluorescence image. Scale bars are 100 µm.

(C) The pips_h sub-cell-line from picked RFP positive clones. Left, bright filed image. Right, red fluorescence image. Scale bars are 100 µm.
